# Advances in the Development of Microbial Double-Stranded RNA Production Systems for Application of RNA Interference in Agricultural Pest Control

**DOI:** 10.3389/fbioe.2021.753790

**Published:** 2021-09-13

**Authors:** Ruobing Guan, Dongdong Chu, Xinyi Han, Xuexia Miao, Haichao Li

**Affiliations:** ^1^ State Key Laboratory of Wheat and Maize Crop Science/College of Plant Protection, Henan Agricultural University, Zhengzhou, China; ^2^ Key Laboratory of Insect Developmental and Evolutionary Biology, CAS Center for Excellence in Molecular Plant Sciences, Shanghai Institute of Plant Physiology and Ecology, Innovation Academy for Seed Design, Chinese Academy of Sciences, Shanghai, China

**Keywords:** RNA interference, dsRNA, microbes, synthetic biology, production

## Abstract

RNA interference (RNAi) is a valuable and revolutionary technology that has been widely applied in medicine and agriculture. The application of RNAi in various industries requires large amounts of low-cost double-stranded RNA (dsRNA). Chemical synthesis can only produce short dsRNAs; long dsRNAs need to be synthesized biologically. Several microbial chassis cells, such as *Escherichia coli*, *Saccharomyces cerevisiae*, and *Bacillus* species, have been used for dsRNA synthesis. However, the titer, rate of production, and yield of dsRNA obtained by these microorganism-based strategies is still low. In this review, we summarize advances in microbial dsRNA production, and analyze the merits and faults of different microbial dsRNA production systems. This review provides a guide for dsRNA production system selection. Future development of efficient microbial dsRNA production systems is also discussed.

## Introduction

The large-scale use of chemical pesticides creates tremendous ecological pressure on soil, water, air, and the human living environment. After the long-term use of chemical pesticides, resistance, resurgence, and residue (3R) problems have become increasingly prominent (Tudi et al., 2021). The emergence of RNA interference (RNAi) technology has brought new hope of solving these problems. In this technology, double-stranded RNA (dsRNA) enters a host and triggers the RNAi effect—the expression of the complementary target gene is silenced, which affects the growth and development of the target organism, thus achieving pest control (Fire et al., 1998; Fletcher et al., 2020; Zhu and Palli, 2020). RNAi pesticides are considered novel, and ecofriendly, because RNAi technology uses precise targeting and the pesticide agent could be easily degradable.

However, several problems need to be solved before this technology can be widely applied, such as efficient, high-throughput target gene acquisition, dsRNA delivery strategies in different organisms (insects, plants, fungi, bacteria, and viruses), the stability of the dsRNA in field application, and construction of multi-species integrated control strategies in complex ecological environments (Zhang et al., 2013). Besides, large-scale, low-cost synthesis of dsRNA is crucial for applying RNAi technology in agriculture (Silver et al., 2021). Chemical synthesis of RNA is suitable for the synthesis of short RNAs, such as small interfering RNAs (siRNAs), because the synthesis error rate increases and the yield decreases when the length of the target RNA product increases (Mu et al., 2018). *In vitro* synthesis strategies relying on T7/SP6 RNA polymerase and *in vivo* synthesis by engineered bacteria are often used for dsRNA synthesis. The *in vitro* synthesis strategy can produce high-purity dsRNA, but the cost is relatively high. Moreover, this method requires auxiliary materials, such as DNA templates, enzymes, and nucleotides (Mu et al., 2018). The *in vivo* synthesis strategy produces low-cost dsRNA in high yields, but this strategy requires later purification of the product and inactivation of the engineered microbial strain (Mendiola et al., 2020). Nevertheless, the *in vivo* synthesis strategy is more likely to reduce dsRNA production costs and increase yields in the future (Cooper et al., 2021). In this review, applications of microbe-mediated dsRNA expression systems are summarized, and the selection of efficient microbial dsRNA production systems is discussed.

### *Escherichia coli* dsRNA Expression Systems

*E. coli* is a commonly used bacterium for dsRNA expression because of its clear genetic background and convenient genetic manipulation. *E. coli* strain HT115 (DE3), which is RNase III deficient, and L4440 vector with a pair of oppositely oriented T7 promoters (one on each side of the multiple cloning site) are widely used as an expression strain and vector for dsRNA production (Timmons et al., 2001; Voloudakis et al., 2015). After introduction of the L4440 vector ligated with the target fragment into strain HT115 (DE3), large amounts of T7 RNA polymerase can be synthesized on induction by isopropyl β-D-1-thiogalactopyranoside (IPTG); the T7 RNA polymerase binds to the T7 promoter in L4440, which mediates the transcription of downstream DNA sequences into RNA. As a result, two complementary RNAs are synthesized, which in turn form the target dsRNA (Voloudakis et al., 2015).

The production of dsRNA using engineered bacterial expression was first attempted by Timmons and Fire (1998), and the corresponding RNAi phenotype was verified after feeding to the nematode *Caenorhabditis elegans*, showing that dsRNA expressed by bacteria can induce RNAi effects (Timmons et al., 2001). Using this dsRNA generation strategy, RNAi effects were induced in a variety of insects, such as *Spodoptera exigua* (Tian et al., 2009), *Bactrocera dorsalis* (Li et al., 2011), *Chilo infuscatellus* (Zhang et al., 2012), *Spodoptera exigua* (Vatanparast and Kim, 2017), *Plagiodera versicolora* (Zhang et al., 2019), *Tuta absoluta* (Bento et al., 2020), *Harmonia axyridis* (Ma et al., 2020), *Spodoptera littoralis* (Caccia et al., 2020), *Agrilus planipennis* (Leelesh and Rieske, 2020). In addition, expressed virus dsRNA can protect a plant or animal against viral infection. For example, *E. coli* strain HT115 (DE3) was used to express dsRNA of the Chinese Sacbrood Virus (CSBV) VP1, which was fed to Chinese honeybees (*Apis cerana*) and effectively prevented the virus infecting the bees (Zhang et al., 2016). Treating *Nicotiana benthamiana* with dsRNAs of fragments of two major plant viruses, Pepper Mild Mottle Virus (PMMoV) and Plum Pox Virus (PPV), effectively reduced the infection of *N. benthamiana* by these two viruses (Tenllado et al., 2003). All these results show that engineered *E. coli* can synthesize dsRNAs, and the dsRNA produced can induce RNAi effects in the corresponding target organisms.

The yield of dsRNA synthesized in *E. coli* has been improved over time. An average of 4 μg of dsRNA was obtained per ml of *E. coli* culture in 2003 (Tenllado et al., 2003), and 45 μg hairpin dsRNA per ml of bacteria (optical density at 600 nm = 1) in 2013 (Posiri et al., 2013). The increase of dsRNA yield is due to the fermentation methods and operation parameters used (Thammasorn et al., 2015; Papic et al., 2018). dsRNA production using batch fermentation and fed-batch fermentation was compared in a 10 L fermenter, and the dsRNA titer in fed-batch fermentation (95.0 ± 21.5 μg/ml) was nearly 30-fold that found in batch fermentation (3.4 ± 0.5 μg/ml) (Thammasorn et al., 2015). The nutrition can also affect the final dsRNA yield, and the production of dsRNA using Terrific broth (TB) (6.2 ± 0.2 μg/ml) was higher than that using Luria-Bertani (LB) broth (2.6 ± 0.8 μg/ml). After further optimization, the yield was close to 0.06 g/g, the maximum production rate reached 11.1 mg L^−1^ h^−1^ by batch fermentation and 15.2 mg L^−1^ h^−1^ by fed-batch fermentation (Papic et al., 2018). Therefore, the dsRNA yield is related to bacterial growth, and fed-batch fermentation resulted in a higher dsRNA yield by sustainably supplying nutrition.

Modification of the expression vector and host strain can further improve the efficiency of dsRNA synthesis. dsRNA production using a new *E. coli* expression system, pET28-BL21 (DE3) RNase III- was thrice than that of L4440-HT115 (DE3) (Ma et al., 2020).

Moreover, extraction methods are closely linked to the yield of dsRNA. The titer of dsRNA extracted from *E. coli* by ultrasonic crushing and phenol extraction was 19.5 μg/ml, while sonication and heating before dsRNA extraction increased the titer of dsRNA by 2.5- to 5- fold (Ahn et al., 2019).

Nowadays, large-scale synthesis of dsRNA in *E. coli* has developed, but further increasing the titer, rate, and yield (TRY) of dsRNA production is essential for future applications.

### *Saccharomyces cerevisiae* dsRNA Expression Systems

The model eukaryotic species *Saccharomyces cerevisiae* has also been used as a chassis for dsRNA production. *S. cerevisiae* has a clear genetic background, easy genetic engineering methods, and well-developed fermentation processes (Nandy and Srivastava, 2018). Besides, *S. cerevisiae* does not contain the core genes *Dicer-2* and *Argonaute-2* of the RNAi pathway (Drinnenberg et al., 2009), which allows efficient dsRNA synthesis in *S. cerevisiae* compared with *E. coli* and other bacterial species (Zhong et al., 2019). Similarly, plant chloroplast does not contain RNAi pathway, and dsRNA can be enriched to 0.4% of total RNA in plant chloroplast; expression of dsRNA in plant chloroplast can be used to protect plants from being fed by insects, which would be more efficient than expressing dsRNA form the plant leaves (Zhang et al., 2015).

Feeding the fruit fly *Drosophila suzukii* with recombinant yeast expressing insect dsRNA targeting *y-Tubulin* resulted in a significant reduction in larval survivorship, adult motility, and reproduction (Murphy et al., 2016). Moreover, feeding *D. suzukii* with genetically modified *S. cerevisiae* expressing dsRNA (targeting *y-tubulin23C*) resulted in a significant decrease in the fitness of *D. suzukii* in the environment (Abrieux and Chiu, 2016).

The expression of dsRNA in *S. cerevisiae* has also been validated in the mosquito *Aedes aegypti*. *Fez2* and *lrc* were selected as target genes in *A. aegypti*, and shRNAs of these genes were expressed in *S. cerevisiae*. When the genetically-modified *S. cerevisiae* was heated, dried, and fed to insects, this led to >95%mortality of *A. aegypti* (Hapairai et al., 2017). The same effects were observed with *Aedes albopictus*, *Anopheles gambiae*, and *Culex quinquefasciatus* (Mysore et al., 2017; Mysore et al., 2019a; Mysore et al., 2019b). In this way, biocontrol strategies for specific mosquito species can be developed, to effectively suppress human diseases transmitted by mosquitoes.

Many insects, livestock, aquaculture species and humans consume yeast. Therefore, developing efficient yeast expression systems might increase the possibility of applying yeast-derived dsRNA commercially (Duman-Scheel, 2019). The dsRNA produced by *S. cerevisiae* can also serve as a potential oral delivery system for shRNA to mammalian cells (mouse intestinal DCs) and be used in human disease therapeutics (Zhang et al., 2014; Duman-Scheel, 2019). Several companies have developed yeast dsRNA expression systems. In May 2019, Renaissance BioScience filed a patent application for the production and delivery of bioactive dsRNA ingredients using yeasts.

In the future, large-scale production of dsRNA in *S. cerevisiae* can be enhanced by improving the expression vectors (Crook et al., 2014), the promoters for the dsRNA transcription (Voineagu et al., 2008), the length of the hairpins (Yoshimatsu and Nagawa, 1989), and the sites of integration positions (Kim et al., 2015).

### *Bacillus* dsRNA Expression Systems

Some *B. subtilis* strains are classified as probiotics for human and animal consumption (Rosales-Mendoza and Angulo, 2015). Therefore, this species has also been selected for dsRNA expression. The dsRNA (*daf-2*, *unc-62*) expression vector pBSR was introduced into *B. subtilis*, and feeding *C. elegans* this genetically-modified *B. subtilis* strain induced RNAi effects (Lezzerini et al., 2015). A *B. subtilis* strain with dsVP28 expression was able to effectively prevent shrimp infection with white spot syndrome virus (WSSV); the survival rate of shrimp treated with the *B. subtilis* strain was 91.67%, while that in the control group was only 28.57% (Saelim et al., 2020).

*Bacillus thuringiensis* (Bt) is an effective biopesticide production strain that has been widely used for control of lepidopteran pests. Bt has been used as an expression host for dsRNA production. In the vector pBTdsSBV-VP1, two spore-producing-dependent cyt1Aa promoters in opposite direction were linked to the *VP1* gene of Sacbrood virus (SBV), and a Shine-Dalgarno sequence (GAAAGGAGG) was added at specific positions, which increased the stability of the RNA. Transfer of pBTdsSBV-VP1 into Bt strain 4Q7 led to the expression of dsRNA. Feeding the total RNA extracted from this Bt strain to *Apis cerana* (honeybees) infected with SBV virus significantly reduced the viral infection of the insects (Park et al., 2020).

The Bt-based dsRNA production platform has some advantages compared with other platforms. The *cry* sporulation-dependent gene promotor was used for dsRNA expression, and the dsRNA could be produced during the sporulation phase of Bt. Moreover, other expression systems (like *E. coli*, *B. subtiis*, *S. cerevisiae* expression systems) require an inducer (IPTG or others) to induce dsRNA expression, but no inducer is needed for expression in Bt. Finally, Bt cells can undergo enzyme-associated autolysis after sporulation, thus cell lysis is not required for dsRNA extraction (Park et al., 2020).

With the increase of insect resistance to Bt, the use of Bt as a platform for dsRNA expression would help with pest control *via* a Bt + RNAi strategy (Caccia et al., 2020; Kang et al., 2021). Therefore, the Bt dsRNA expression system could be a useful dsRNA production platform for the introduction of RNAi in organisms.

### Insect-Symbiotic Bacteria dsRNA Expression Systems

There are abundant symbiotic bacteria in the oral tract and gut of insects, and they interact directly with the insects and plants. Some symbiotic bacteria can easily be genetically manipulated, so they might be potentially efficient dsRNA production platforms for insect control. The use of insect-symbiotic bacteria to express dsRNA for insect control is known as symbiont-mediated RNAi (SMR) (Taracena et al., 2015; Whitten et al., 2016; Whitten and Dyson, 2017; Whitten, 2019; Asgari et al., 2020).

*Rhodococcus rhodnii* (*R. rhodnii*), a symbiotic bacterium of the triatomine *Rhodnius prolixus*, was used to express RHBP-specific hairpin RNA; the gene expression products of RHBP can suppress *R. prolixus* by affecting its adult oviposition (Taracena et al., 2015). Subsequently, two symbiotic bacterial strains, *R. rhodnii* and BFo2 (a member of the *Enterobacteriales*), were isolated from the insects *R. prolixus* and *Frankliniella occidentalis* (western flower thrips), respectively. The RNase III gene was knocked out and dsRNA expression cassettes was expressed in these two insect symbiotic bacteria; when the engineered bacteria were taken up by insects, the dsRNA functioned in the hosts, inducing RNAi effects (Whitten et al., 2016).

*Snodgrassella alvi*, a core gut symbiotic bacterium of the honeybee *A. mellifera*, was modified as a dsRNA-producing host. The dsRNA produced by the engineered *S. alvi* can suppress gene expression in *A. mellifera*. Moreover, this dsRNA can suppress genes of parasitic *Varroa* mites and kill them, which protects the honey-bees from the *Varroa* mites, the most threatening pest to the world’s beekeeping industry (Leonard et al., 2020). Based on this technology, a new bioproduct, “BioDirect” was registered as dsRNA for the prevention and control of *Varroa mites*. This is the first dsRNA biopesticide active ingredient submitted to the U.S. Environmental Protection Agency (EPA) for exogenous application in agriculture.

Thus, SMR is not only potential pest control agents, but can also be beneficial for insect protection. SMR depends on both the specificity of RNAi toward the targeted insect gene, and the specificity of the symbiotic bacterium for its host. This dual specificity makes SMR a precision control tool, and this tool is obviously different from chemical insecticides. However, there are issues that need to be addressed before symbiotic bacteria can reliably serve as dsRNA expression hosts. The first is to find suitable symbiotic bacteria that stably colonize the host insects; the bacterial content should also be relatively high in the host insect. The second is that the symbiotic bacteria must be able to express dsRNA efficiently and stably. Thus, the acquisition and modification of symbiotic bacteria and colonization of the engineered symbiotic bacteria in the host need to be addressed before applying SMR for dsRNA production.

Nevertheless, this SMR strategy is specific for pest control without increasing environmental stress, and it might be widely used in the future.

## Conclusion and Perspectives

Genetic engineering of microorganisms for large-scale production of dsRNA is feasible. Currently, *E. coli*, *Bacillus, S. cerevisiae* and several other symbiotic bacteria have mature expression systems for dsRNA production. As most of these bacteria are probiotics and/or model species, they might be the most suitable microbial hosts for diverse dsRNA production. *Corynebacterium glutamicum* has also been shown to efficiently synthesize dsRNA longer than 1 kbp in a yield >1 g/L of culture (Hashero et al., 2021). Besides, microalgae can also be engineered as dsRNA expression vectors, and shrimps and crabs can be protected from bacterial or viral infection by feeding on microalgae expressing dsRNA (Saksmerprome et al., 2009; Somchai et al., 2016; Charoonnart et al., 2019). Fungi (Chen et al., 2015) and viruses (Dubreuil et al., 2009; Kumar et al., 2012) have also been engineered to produce dsRNA, and better results have been obtained.

**FIGURE 1:**
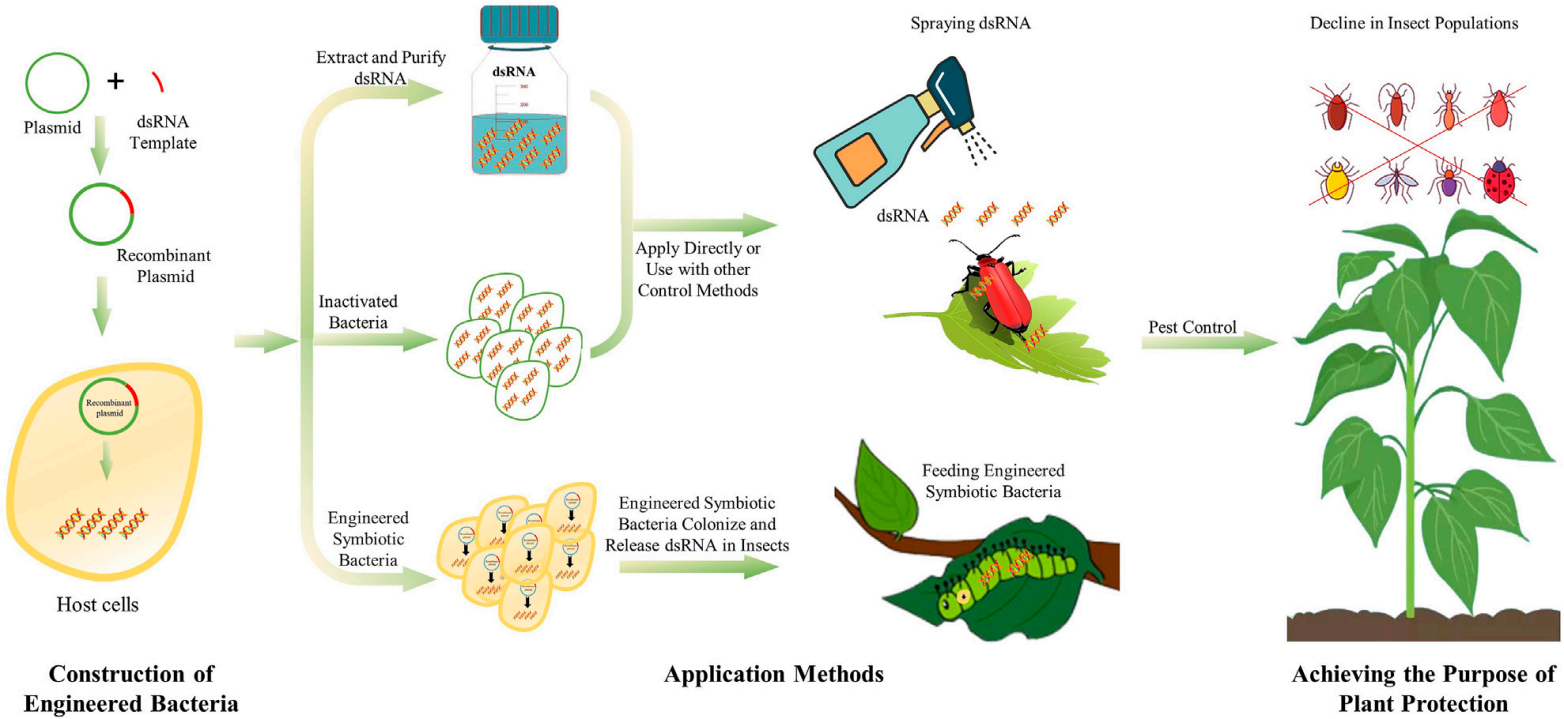
The process and use method of microbial dsRNA production system.

The dsRNA synthesized by microbes can be used directly in live or inactivated microbes. However, engineered microbes entering a host induce immune responses, which might compromise the desired RNAi effects. Moreover, the engineered microbes may spread into the environment, and lead to sustainable expression of dsRNA, which might affect non-target species in the environment. Besides, plasmid-based expression elements may be transferred inter-species, resulting in biological contamination problems (Mendelsohn et al., 2020). dsRNA produced in engineered bacteria cannot be secreted directly outside the cell. Therefore, lysis, extraction and purification are required to obtained dsRNA production. The lysis of cells can be performed by ultrasonication, enzymatic lysis, boiling lysis, while sodium dodecyl sulfate (SDS) can be used to enhance the lysis (Posiri et al., 2013). After the cell wall is broken, the nucleic acid can be released to obtain a crude extract of dsRNA. Then, use appropriate RNA extraction methods, such as TRIzol reagent or other RNA extraction reagents, to obtain pure dsRNA production. Extracting and purifying dsRNA from engineered bacteria will avoid or reduce the problems mentioned above. However, these processes are relatively cumbersome and need to be further optimized. The dsRNA obtained through microbial-production can be directly applied to pest control by spraying, and the nanocarrier-mediated transdermal dsRNA delivery system can facilitate the development of sprayable RNA pesticides (Zheng et al., 2019; Yan et al., 2020). Which method to use also needs to be selected according to different environments (Figure 1).

There are also some further technical issues in this field that need to be solved. For example, substrates for industrial fermentation can be contaminated with various bacteria, and such contaminants can inhibit growth of the desired (dsRNA-expressing) bacteria and reduce the efficiency of the fermentation process, thus significantly reducing productivity (Seo et al., 2020). Antimicrobial decontamination strategies have been developed, but the metabolites produced and antibiotics used to avoid contamination by other microorganisms are released, inevitably putting pressure on the environment and increasing risks to human health (Kraemer et al., 2019). Once these problems are solved and dsRNAs can be produced by large-scale fermentation, they will have broad application prospects and bring huge economic benefits.

dsRNA production methods have been continuously optimized in recent years to adapt to production needs and promote the application of this technology. The cost of dsRNA was approximately US$12,000/g in 2008, dropping to US$60/g in 2018, and in 2020, RNAGri had the ability to produce tons of dsRNA at a cost of US$1/g, Greenlight’s GreenWorX^™^ system can further reduce the cost of dsRNA synthesis to < US$0.5/g (Cagliari et al., 2019; Suhag et al., 2020; Taning et al., 2020), which will provide material for the economical large-scale application of dsRNA-based pesticides.

The large-scale application of RNAi technology relies on the construction of efficient and appropriate microbial cell factories for dsRNA production. With the development of synthetic biology, global rewiring of the expression systems of model species to increase dsRNA expression levels will be possible. In the future, active engineered microorganisms for dsRNA production and low-cost purified dsRNA will become available leading to greener agriculture without chemical pesticides to protect plants from insects and microbial infections.

## References

[B1] Abrieux A. Chiu J. C. (2016). Oral Delivery of dsRNA by Microbes: Beyond Pest Control. Communicative Integr. Biol. 9, e1236163. 10.1080/19420889.2016.1236163 PMC5193050 28042376

[B2] Ahn S. J. Donahue K. Koh Y. Martin R. R. Choi M. Y. (2019). Microbial-Based Double-Stranded RNA Production to Develop Cost-Effective RNA Interference Application for Insect Pest Management. Int. J. Insect Sci. 11, 1179543319840323. 10.1177/1179543319840323 31040730 PMC6482651

[B3] Asgari M. Ilbeigikhamsehnejad M. Rismani E. Dinparast Djadid N. Raz A. (2020). Molecular Characterization of RNase III Protein of Asaia Sp. For Developing a Robust RNAi-Based Paratransgensis Tool to Affect the Sexual Life-Cycle of Plasmodium or Anopheles Fitness. Parasites Vectors 13, 42. 10.1186/s13071-020-3889-6 31996254 PMC6990573

[B4] Bento F. M. Marques R. N. Campana F. B. Demétrio C. G. Leandro R. A. Parra J. R. P. (2020). Gene Silencing by RNAi *via* Oral Delivery of dsRNA by Bacteria in the South American Tomato Pinworm, Tuta Absoluta. Pest Manag. Sci. 76, 287–295. 10.1002/ps.5513 31207074

[B5] Caccia S. Astarita F. Barra E. Di Lelio I. Varricchio P. Pennacchio F. (2020). Enhancement of Bacillus Thuringiensis Toxicity by Feeding Spodoptera Littoralis Larvae with Bacteria Expressing Immune Suppressive dsRNA. J. Pest Sci. 93, 303–314. 10.1007/s10340-019-01140-6

[B6] Cagliari D. Dias N. P. Galdeano D. M. Dos Santos E. Á. Smagghe G. Zotti M. J. (2019). Management of Pest Insects and Plant Diseases by Non-Transformative RNAi. Front. Plant Sci. 10, 1319. 10.3389/fpls.2019.01319 31708946 PMC6823229

[B7] Charoonnart P. Worakajit N. Zedler J. A. Z. Meetam M. Robinson C. Saksmerprome V. (2019). Generation of Microalga Chlamydomonas Reinhardtii Expressing Shrimp Antiviral dsRNA without Supplementation of Antibiotics. Sci. Rep. 9, 3164. 10.1038/s41598-019-39539-x 30816201 PMC6395707

[B8] Chen X. Li L. Hu Q. Zhang B. Wu W. Jin F. (2015). Expression of dsRNA in Recombinant Isaria Fumosorosea Strain Targets the TLR7 Gene in Bemisia Tabaci. BMC Biotechnol. 15, 64. 10.1186/s12896-015-0170-8 26198409 PMC4509747

[B9] Cooper A. M. Song H. Yu Z. Biondi M. Bai J. Shi X. (2021). Comparison of Strategies for Enhancing RNA Interference Efficiency in Ostrinia Nubilalis. Pest Manag. Sci. 77, 635–645. 10.1002/ps.6114 33002336 PMC7855606

[B10] Crook N. C. Schmitz A. C. Alper H. S. (2014). Optimization of a Yeast RNA Interference System for Controlling Gene Expression and Enabling Rapid Metabolic Engineering. ACS Synth. Biol. 3, 307–313. 10.1021/sb4001432 24328131

[B11] Drinnenberg I. A. Weinberg D. E. Xie K. T. Mower J. P. Wolfe K. H. Fink G. R. (2009). RNAi in Budding Yeast. Science 326, 544–550. 10.1126/science.1176945 19745116 PMC3786161

[B12] Dubreuil G. Magliano M. Dubrana M. P. Lozano J. Lecomte P. Favery B. (2009). Tobacco Rattle Virus Mediates Gene Silencing in a Plant Parasitic Root-Knot Nematode. J. Exp. Bot. 60, 4041–4050. 10.1093/jxb/erp237 19625337

[B13] Duman-Scheel M. (2019). *Saccharomyces cerevisiae* (Baker's Yeast) as an Interfering RNA Expression and Delivery System. Curr. Drug Targets 20, 942–952. 10.2174/1389450120666181126123538 30474529 PMC6700756

[B14] Fire A. Xu S. Montgomery M. K. Kostas S. A. Driver S. E. Mello C. C. (1998). Potent and Specific Genetic Interference by Double-Stranded RNA in *Caenorhabditis elegans* . Nature 391, 806–811. 10.1038/35888 9486653

[B15] Fletcher S. J. Reeves P. T. Hoang B. T. Mitter N. (2020). A Perspective on RNAi-Based Biopesticides. Front. Plant Sci. 11, 51. 10.3389/fpls.2020.00051 32117388 PMC7028687

[B16] Hapairai L. K. Mysore K. Chen Y. Harper E. I. Scheel M. P. Lesnik A. M. (2017). Lure-and-Kill Yeast Interfering RNA Larvicides Targeting Neural Genes in the Human Disease Vector Mosquito *Aedes aegypti* . Sci. Rep. 7, 13223. 10.1038/s41598-017-13566-y 29038510 PMC5643370

[B17] Hashiro S. Chikami Y. Kawaguchi H. Krylov A. A. Niimi T. Yasueda H. (2021). Efficient Production of Long Double-Stranded RNAs Applicable to Agricultural Pest Control by *Corynebacterium Glutamicum* Equipped with Coliphage T7-Expression System. Appl. Microbiol. Biotechnol. 105, 4987–5000. 10.1007/s00253-021-11324-9 34097118 PMC8236056

[B18] Kang S. Sun D. Qin J. Guo L. Zhu L. Bai Y. (2021). Fused: A Promising Molecular Target for an RNAi-Based Strategy to Manage Bt Resistance in Plutella Xylostella (L.). J. Pest Sci. 10.1007/s10340-021-01374-3

[B19] Kim H. Yoo S. J. Kang H. A. (2015). Editorial: Yeast Synthetic Biology: New Tools to Unlock Cellular Function. FEMS Yeast Res. 15, 1. 10.1093/femsyr/fou003 27926475

[B20] Kraemer S. A. Ramachandran A. Perron G. G. (2019). Antibiotic Pollution in the Environment: From Microbial Ecology to Public Policy. Microorganisms 7, 180. 10.3390/microorganisms7060180 PMC6616856 31234491

[B21] Kumar P. Pandit S. S. Baldwin I. T. (2012). Tobacco Rattle Virus Vector: A Rapid and Transient Means of Silencing Manduca Sexta Genes by Plant Mediated RNA Interference. PLoS One 7, e31347. 10.1371/journal.pone.0031347 22312445 PMC3270032

[B22] Leelesh R. S. Rieske L. K. (2020). Oral Ingestion of Bacterially Expressed dsRNA Can Silence Genes and Cause Mortality in a Highly Invasive, Tree-Killing Pest, the Emerald Ash Borer. Insects 11, 440. 10.3390/insects11070440 PMC7411747 32674291

[B23] Leonard S. P. Powell J. E. Perutka J. Geng P. Heckmann L. C. Horak R. D. (2020). Engineered Symbionts Activate Honey Bee Immunity and Limit Pathogens. Science 367, 573–576. 10.1126/science.aax9039 32001655 PMC7556694

[B24] Lezzerini M. Van De Ven K. Veerman M. Brul S. Budovskaya Y. V. (2015). Specific RNA Interference in *Caenorhabditis elegans* by Ingested dsRNA Expressed in Bacillus Subtilis. PLoS One 10, e0124508. 10.1371/journal.pone.0124508 25928543 PMC4416053

[B25] Li X. Zhang M. Zhang H. (2011). RNA Interference of Four Genes in Adult Bactrocera Dorsalis by Feeding Their dsRNAs. PLoS One 6, e17788. 10.1371/journal.pone.0017788 21445257 PMC3060817

[B26] Ma Z. Z. Zhou H. Wei Y. L. Yan S. Shen J. (2020). A Novel Plasmid- *Escherichia coli* System Produces Large Batch dsRNAs for Insect Gene Silencing. Pest Manag. Sci. 76, 2505–2512. 10.1002/ps.5792 32077251

[B27] Mendelsohn M. L. Gathmann A. Kardassi D. Sachana M. Hopwood E. M. Dietz-Pfeilstetter A. (2020). Summary of Discussions from the 2019 OECD Conference on RNAi Based Pesticides. Front. Plant Sci. 11, 740. 10.3389/fpls.2020.00740 32547591 PMC7274041

[B28] Mendiola S. Y. Civitello D. J. Gerardo N. M. (2020). An Integrative Approach to Symbiont-Mediated Vector Control for Agricultural Pathogens. Curr. Opin. Insect Sci. 39, 57–62. 10.1016/j.cois.2020.02.007 32299043

[B29] Mu X. Greenwald E. Ahmad S. Hur S. (2018). An Origin of the Immunogenicity of *In Vitro* Transcribed RNA. Nucleic Acids Res. 46, 5239–5249. 10.1093/nar/gky177 29534222 PMC6007322

[B30] Murphy K. A. Tabuloc C. A. Cervantes K. R. Chiu J. C. (2016). Ingestion of Genetically Modified Yeast Symbiont Reduces Fitness of an Insect Pest *via* RNA Interference. Sci. Rep. 6, 22587. 10.1038/srep22587 26931800 PMC4773866

[B31] Mysore K. Hapairai L. K. Sun L. Harper E. I. Chen Y. Eggleson K. K. (2017). Yeast Interfering RNA Larvicides Targeting Neural Genes Induce High Rates of Anopheles Larval Mortality. Malar. J. 16, 461. 10.1186/s12936-017-2112-5 29132374 PMC5683233

[B32] Mysore K. Hapairai L. K. Wei N. Realey J. S. Scheel N. D. Severson D. W. (2019a). Preparation and Use of a Yeast shRNA Delivery System for Gene Silencing in Mosquito Larvae. Methods Mol. Biol. 1858, 213–231. 10.1007/978-1-4939-8775-7_15 30414120 PMC7202713

[B33] Mysore K. Li P. Wang C.-W. Hapairai L. K. Scheel N. D. Realey J. S. (2019b). Characterization of a Broad-Based Mosquito Yeast Interfering RNA Larvicide with a Conserved Target Site in Mosquito Semaphorin-1a Genes. Parasites Vectors 12, 256. 10.1186/s13071-019-3504-x 31118082 PMC6532267

[B34] Nandy S. K. Srivastava R. K. (2018). A Review on Sustainable Yeast Biotechnological Processes and Applications. Microbiol. Res. 207, 83–90. 10.1016/j.micres.2017.11.013 29458873

[B35] Papić L. Rivas J. Toledo S. Romero J. (2018). Double-stranded RNA Production and the Kinetics of Recombinant *Escherichia coli* HT115 in Fed-Batch Culture. Biotechnol. Rep. (Amst) 20, e00292. 10.1016/j.btre.2018.e00292 30568886 PMC6288044

[B36] Park M. G. Kim W. J. Choi J. Y. Kim J. H. Park D. H. Kim J. Y. (2020). Development of a Bacillus Thuringiensis Based dsRNA Production Platform to Control Sacbrood Virus in *Apis cerana* . Pest Manag. Sci. 76, 1699–1704. 10.1002/ps.5692 31758591

[B37] Posiri P. Ongvarrasopone C. Panyim S. (2013). A Simple One-Step Method for Producing dsRNA from *E. coli* to Inhibit Shrimp Virus Replication. J. Virol. Methods 188, 64–69. 10.1016/j.jviromet.2012.11.033 23247053

[B38] Rosales-Mendoza S. Angulo C. (2015). Bacillus Subtilis Comes of Age as a Vaccine Production Host and Delivery Vehicle. Expert Rev. Vaccin. 14, 1135–1148. 10.1586/14760584.2015.1051469 26028252

[B39] Saelim H. Loprasert S. Phongdara A. (2020). Bacillus Subtilis Expressing dsVP28 Improved Shrimp Survival from WSSV challenge. ScienceAsia 46S, 19. 10.2306/scienceasia1513-1874.2020.s003

[B40] Saksmerprome V. Charoonnart P. Gangnonngiw W. Withyachumnarnkul B. (2009). A Novel and Inexpensive Application of RNAi Technology to Protect Shrimp from Viral Disease. J. Virol. Methods 162, 213–217. 10.1016/j.jviromet.2009.08.010 19712700

[B41] Seo S. O. Park S. K. Jung S. C. Ryu C. M. Kim J. S. (2020). Anti-Contamination Strategies for Yeast Fermentations. Microorganisms 8, 274. 10.3390/microorganisms8020274 PMC7074673 32085437

[B42] Silver K. Cooper A. M. Zhu K. Y. (2021). Strategies for Enhancing the Efficiency of RNA Interference in Insects. Pest Manag. Sci. 77, 2645–2658. 10.1002/ps.6277 33440063

[B43] Somchai P. Jitrakorn S. Thitamadee S. Meetam M. Saksmerprome V. (2016). Use of Microalgae Chlamydomonas Reinhardtii for Production of Double-Stranded RNA against Shrimp Virus. Aquacult. Rep. 3, 178–183. 10.1016/j.aqrep.2016.03.003

[B44] Suhag A. Yadav H. Chaudhary D. Subramanian S. Jaiwal R. Jaiwal P. K. (2020). Biotechnological Interventions for the Sustainable Management of a Global Pest, Whitefly (Bemisia Tabaci). Insect Sci. 10.1111/1744-7917.12853 32696581

[B45] Taning C. N. Arpaia S. Christiaens O. Dietz‐Pfeilstetter A. Jones H. Mezzetti B. (2020). RNA‐Based Biocontrol Compounds: Current Status and Perspectives to Reach the Market. Pest Manag. Sci. 76, 841–845. 10.1002/ps.5686 31743573

[B46] Taracena M. L. Oliveira P. L. Almendares O. Umaña C. Lowenberger C. Dotson E. M. (2015). Genetically Modifying the Insect Gut Microbiota to Control Chagas Disease Vectors through Systemic RNAi. Plos Negl. Trop. Dis. 9, e0003358. 10.1371/journal.pntd.0003358 25675102 PMC4326462

[B47] Tenllado F. Martínez-García B. Vargas M. Díaz-Ruíz J. R. (2003). Crude Extracts of Bacterially Expressed dsRNA Can Be Used to Protect Plants against Virus Infections. BMC Biotechnol. 3, 3. 10.1186/1472-6750-3-3 12659646 PMC153545

[B48] Thammasorn T. Sangsuriya P. Meemetta W. Senapin S. Jitrakorn S. Rattanarojpong T. (2015). Large-Scale Production and Antiviral Efficacy of Multi-Target Double-Stranded RNA for the Prevention of white Spot Syndrome Virus (WSSV) in Shrimp. BMC Biotechnol. 15, 110. 10.1186/s12896-015-0226-9 26626024 PMC4667486

[B49] Tian H. Peng H. Yao Q. Chen H. Xie Q. Tang B. (2009). Developmental Control of a Lepidopteran Pest Spodoptera Exigua by Ingestion of Bacteria Expressing dsRNA of a Non-Midgut Gene. PLoS One 4, e6225. 10.1371/journal.pone.0006225 19593438 PMC2704864

[B50] Timmons L. Court D. L. Fire A. (2001). Ingestion of Bacterially Expressed dsRNAs Can Produce Specific and Potent Genetic Interference in *Caenorhabditis elegans* . Gene. 263, 103–112. 10.1016/s0378-1119(00)00579-5 11223248

[B51] Timmons L. Fire A. (1998). Specific Interference by Ingested dsRNA. Nature 395, 854. 10.1038/27579 9804418

[B52] Tudi M. Daniel Ruan H. Wang L. Lyu J. Sadler R. Connell D. (2021). Agriculture Development, Pesticide Application and its Impact on the Environment. Int. J. Environ. Res. Public Health 18, 1112. 10.3390/ijerph18031112 33513796 PMC7908628

[B53] Vatanparast M. Kim Y. (2017). Optimization of Recombinant Bacteria Expressing dsRNA to Enhance Insecticidal Activity against a Lepidopteran Insect, Spodoptera Exigua. PLoS One 12, e0183054. 10.1371/journal.pone.0183054 28800614 PMC5553977

[B54] Voineagu I. Narayanan V. Lobachev K. S. Mirkin S. M. (2008). Replication Stalling at Unstable Inverted Repeats: Interplay between DNA Hairpins and fork Stabilizing Proteins. Proc. Natl. Acad. Sci. 105, 9936–9941. 10.1073/pnas.0804510105 18632578 PMC2481305

[B55] Voloudakis A. E. Holeva M. C. Sarin L. P. Bamford D. H. Vargas M. Poranen M. M. (2015). Efficient Double-Stranded RNA Production Methods for Utilization in Plant Virus Control. Methods Mol. Biol. 1236, 255–274. 10.1007/978-1-4939-1743-3_19 25287509

[B56] Whitten M. Dyson P. (2017). Gene Silencing in Non-Model Insects: Overcoming Hurdles Using Symbiotic Bacteria for Trauma-free Sustainable Delivery of RNA Interference: Sustained RNA Interference in Insects Mediated by Symbiotic Bacteria: Applications as a Genetic Tool and as a Biocide. Bioessays 39, 1600247. 10.1002/bies.201600247 28234404

[B57] Whitten M. M. A. Facey P. D. Del Sol R. Fernández-Martínez L. T. Evans M. C. Mitchell J. J. (2016). Symbiont-Mediated RNA Interference in Insects. Proc. R. Soc. B. 283, 20160042. 10.1098/rspb.2016.0042 PMC4810840 26911963

[B58] Whitten M. M. (2019). Novel RNAi Delivery Systems in the Control of Medical and Veterinary Pests. Curr. Opin. Insect Sci. 34, 1–6. 10.1016/j.cois.2019.02.001 31247409 PMC6990399

[B59] Yan S. Qian J. Cai C. Ma Z. Li J. Yin M. (2020). Spray Method Application of Transdermal dsRNA Delivery System for Efficient Gene Silencing and Pest Control on Soybean Aphid *Aphis Glycines* . J. Pest Sci. 93, 449–459. 10.1007/s10340-019-01157-x

[B60] Yoshimatsu T. Nagawa F. (1989). Control of Gene Expression by Artificial Introns in *Saccharomyces cerevisiae* . Science 244, 1346–1348. 10.1126/science.2544026 2544026

[B61] Zhang H. Li H.-C. Miao X.-X. (2013). Feasibility, Limitation and Possible Solutions of RNAi-Based Technology for Insect Pest Control. Insect Sci. 20, 15–30. 10.1111/j.1744-7917.2012.01513.x 23955822

[B62] Zhang J. Khan S. A. Hasse C. Ruf S. Heckel D. G. Bock R. (2015). Full Crop protection from an Insect Pest by Expression of Long Double-Stranded RNAs in Plastids. Science 347, 991–994. 10.1126/science.1261680 25722411

[B63] Zhang J. Zhang Y. Han R. (2016). The High-Throughput Production of dsRNA against Sacbrood Virus for Use in the Honey Bee *Apis cerana* (Hymenoptera: Apidae). Virus Genes 52, 698–705. 10.1007/s11262-016-1346-6 27139728

[B64] Zhang L. Zhang T. Wang L. Shao S. Chen Z. Zhang Z. (2014). *In Vivo* targeted Delivery of CD40 shRNA to Mouse Intestinal Dendritic Cells by Oral Administration of Recombinant Sacchromyces Cerevisiae. Gene Ther. 21, 709–714. 10.1038/gt.2014.50 24871580 PMC4086734

[B65] Zhang Y. Xu L. Li S. Zhang J. (2019). Bacteria-Mediated RNA Interference for Management of Plagiodera Versicolora (Coleoptera: Chrysomelidae). Insects 10, 415. 10.3390/insects10120415 PMC6955681 31766384

[B66] Zhang Y. L. Zhang S. Z. Kulye M. Wu S. R. Yu N. T. Wang J. H. (2012). Silencing of Molt-Regulating Transcription Factor Gene, CiHR3, Affects Growth and Development of Sugarcane Stem Borer, Chilo Infuscatellus. J. Insect Sci. 12, 91. 10.1673/031.012.9101 23427912 PMC3596932

[B67] Zheng Y. Hu Y. Yan S. Zhou H. Song D. Yin M. (2019). A Polymer/Detergent Formulation Improves dsRNA Penetration through the Body wall and RNAi‐Induced Mortality in the Soybean Aphid Aphis Glycines. Pest Manag. Sci. 75, 1993–1999. 10.1002/ps.5313 30610748

[B68] Zhong C. Smith N. A. Zhang D. Goodfellow S. Zhang R. Shan W. (2019). Full-Length Hairpin RNA Accumulates at High Levels in Yeast but Not in Bacteria and Plants. Genes (Basel) 10, 458. 10.3390/genes10060458 PMC6627737 31208028

[B69] Zhu K. Y. Palli S. R. (2020). Mechanisms, Applications, and Challenges of Insect RNA Interference. Annu. Rev. Entomol. 65, 293–311. 10.1146/annurev-ento-011019-025224 31610134 PMC9939233

